# 3D-Printed Hydroxyapatite and Tricalcium Phosphates-Based Scaffolds for Alveolar Bone Regeneration in Animal Models: A Scoping Review

**DOI:** 10.3390/ma15072621

**Published:** 2022-04-02

**Authors:** Nurulhuda Mohd, Masfueh Razali, Mariyam Jameelah Ghazali, Noor Hayaty Abu Kasim

**Affiliations:** 1 Department of Restorative Dentistry, Faculty of Dentistry, Universiti Kebangsaan Malaysia, Jalan Raja Muda Abdul Aziz, Kuala Lumpur 50300, Malaysia; nurulhuda.mohd@ukm.edu.my; 2 Department of Mechanical & Manufacturing Engineering, Faculty of Engineering & Built Environment, Universiti Kebangsaan Malaysia, Bangi 43600, Selangor, Malaysia; mariyam@ukm.edu.my; 3 Faculty of Dentistry, Universiti Kebangsaan Malaysia, Jalan Raja Muda Abdul Aziz, Kuala Lumpur 50300, Malaysia; nhayaty@ukm.edu.my

**Keywords:** 3D printing, biomaterial, bone regeneration, in vivo, hydroxyapatite, tricalcium phosphates

## Abstract

Three-dimensional-printed scaffolds have received greater attention as an attractive option compared to the conventional bone grafts for regeneration of alveolar bone defects. Hydroxyapatite and tricalcium phosphates have been used as biomaterials in the fabrication of 3D-printed scaffolds. This scoping review aimed to evaluate the potential of 3D-printed HA and calcium phosphates-based scaffolds on alveolar bone regeneration in animal models. The systematic search was conducted across four electronic databases: Ovid, Web of Science, PubMed and EBSCOHOST, based on PRISMA-ScR guidelines until November 2021. The inclusion criteria were: (i) animal models undergoing alveolar bone regenerative surgery, (ii) the intervention to regenerate or augment bone using 3D-printed hydroxyapatite or other calcium phosphate scaffolds and (iii) histological and microcomputed tomographic analyses of new bone formation and biological properties of 3D-printed hydroxyapatite or calcium phosphates. A total of ten studies were included in the review. All the studies showed promising results on new bone formation without any inflammatory reactions, regardless of the animal species. In conclusion, hydroxyapatite and tricalcium phosphates are feasible materials for 3D-printed scaffolds for alveolar bone regeneration and demonstrated bone regenerative potential in the oral cavity. However, further research is warranted to determine the scaffold material which mimics the gold standard of care for bone regeneration in the load-bearing areas, including the masticatory load of the oral cavity.

## 1. Introduction

Dental implants have increasingly become the treatment of choice when replacing missing teeth. The key success of the implant therapy is to have an adequate vertical and horizontal bone volume at the implant site [1]. The bone augmentation using particulates bone grafts and titanium mesh has achieved good results in bone reconstruction and implant survival [2]. Thus, placement of the bone grafting materials has become a standard procedure either to augment the atrophic edentulous ridge, minimize bone resorption following extraction of teeth or enhance the healing of osseous defects, with varying rates of success [3]. Alveolar bone augmentation can be carried out by using bone blocks or granulated bone particulates with or without the use of membrane and titanium meshwork, which can be technically challenging, and the success of the treatment will depend on the wound stability and space maintenance of the regenerated defect [4,5,6]. A recent systematic review reported that there is a significant reduction of bone gain when healing complications such as membrane exposure or an abscess occur [7]. Bone regeneration can be achieved using autogenous bone block, which is a gold standard and the most effective graft material for bone regeneration. It is considered superior to the other types of grafts because of the osteogenic, osteoinductive and osteoconductive properties and since it does not produce immunological reactions [8,9]. However, there are associated drawbacks and limitations such as donor site morbidity, extended surgical time and possibly hospital stays, and unavoidable post-operative complications [10,11,12]. In addition, an autogenous bone block is difficult to shape and conform to the osseous defect [13] and the risk of early resorption before bone regeneration takes place, which may compromise the clinical outcome [14]. Alternatively, allografts can be used to eliminate the problem of donor site morbidity and graft quantities [15]. The manufacturing method of allografts has lowered the risk of disease transmission; however, it has been reported that the mechanical and biological properties are affected [15,16]. Xenografts are a graft material of an animal origin which are either freeze-dried or demineralized and deproteinized. The disadvantages associated with xenografts are an increased risk of host immune response and brittleness, even though xenografts have shown to have similar properties to human bone [17].

The effort to address those issues has led to the pursuit of another alternative synthetic bone graft material, which is cost-effective and available in large quantities. Calcium phosphate bioceramic is one of the alloplastic bone grafts that has been routinely used in dental applications for the past decades. Calcium phosphate bioceramics are composed of hydroxyapatite (HA) or tricalcium phosphate (α-TCP and β-TCP), or biphasic calcium phosphate (BCP) which consists of a mixture of HA and β-TCP. Occasionally, bioceramics can be combined to form composite scaffolds to gain higher mechanical properties [18]. Although bone grafts serve as space maintainers and scaffolding, bone substitutes such as allografts, xenografts and alloplastic materials are brittle in nature and unable to conform to the shape of the defects, which eventually may have an impact on the regenerative outcomes. Therefore, it is imperative to develop a novel therapy as an alternative to the standard procedure used for bone regeneration.

Hydroxyapatite (Ca_10_ (PO_4_)_6_ (OH)_2_) (HA) is the most investigated calcium phosphate ceramic compared to other calcium phosphates because it exhibits the same structure, function and chemical composition as bones and teeth. Human bone constitutes up to 70% HA (inorganic component), 25% organic matter and 5% water [19,20]. Hydroxyapatite has been shown to exhibit good cell affinity, which promotes adhesion and proliferation of the osteoblasts and direct bone integration [21,22,23].

In bone tissue engineering, 3D bioprinting is one of the latest technologies which has high precision in fabricating complex tissue structures to mimic bony defects. Stereolithography and micro-extrusion appear to be among the popular techniques for scaffold printing [24,25]. Stereolithography can be used for printing calcium-based bioceramic scaffolds for bone regeneration [26]. Digital light processing (DLP), one of the stereolithography printing systems, can produce biomaterial with a high resolution of 100 μm compared to other 3D printing systems [27]. This technology uses a photocurable ceramic slurry, where each layer-by-layer deposition is cured by light exposure to produce ceramic scaffolds. Thus, 3D bioprinting can create a customized patient-specific design or architecture to perfectly fit the bony defect within a short time [28,29,30]. The 3D scaffolds can enhance cellular attachment, migration, proliferation and osteogenic differentiation for bone regeneration [31]. However, there has been extremely limited clinical trials to validate the efficacy of bone scaffolds tested in animal models. Three-dimensional-printed polycaprolactones (PCL) scaffold has been used in a randomized control trial on socket preservation [32] and a case report of the periodontal osseous defect [33], with varying treatment outcomes, which should be interpreted with caution.

There are several in vivo studies evaluating the use of 3D-printed HA and TCP-based scaffolds in craniofacial bone regeneration involving calvarial bone [18]. However, there is a lack of literature reporting the effect of 3D-printed HA- and TCP-based scaffolds on alveolar bone regeneration or reconstruction of intraoral bony defects. The alveolar bone is a load-bearing structure, and therefore regeneration of intraoral areas is more challenging compared to calvarial bone. The intraoral environment and forces applied during mastication may influence the 3D-printed scaffolds as the space maintainer during the regeneration processes. Therefore, we aimed to conduct a scoping review based on the available literature, identifying the gaps in the quest to develop a new regeneration modality by exploiting 3D printing technology and answering the following questions: (i) How do tissues respond to 3D-printed HA or other calcium phosphate scaffolds during alveolar bone regeneration in animal models? (ii) Are 3D-printed HA or other calcium phosphate scaffolds able to constitute newly regenerated alveolar bone in animal models?

## 2. Materials and Methods

### 2.1. Search Strategy

This review followed the methodology from the Joanna Briggs Institute (JBI) guidelines for scoping reviews and was conducted based on the Preferred Reporting Items for Systematic Reviews and Meta-Analyses extension for Scoping Reviews (PRISMA-ScR) [34,35]. The research questions for the review were: (i) How do tissues respond to 3D-printed HA or other calcium phosphates scaffolds during alveolar bone regeneration in animal models? (ii) Are 3D-printed HA or other calcium phosphate scaffolds able to constitute newly regenerated alveolar bone in animal models?

A search strategy was performed based on the keywords with the following search terms: (“Bone regeneration” OR “Guided bone regeneration”) AND (“3D bioprinting” OR “3D-bioprint*” OR “3-dimensional print*” OR “3D print*” OR “3D-print*” OR “Bioprinting” OR “Three-dimensional bioprint*”) AND (“Hydroxyapatite” OR “Biphasic calcium phosphate” OR “Calcium phosphate*”) AND (“Bone scaffold*” OR “Bone graft*” OR “Synthetic bone*” OR “Bone graft substitute*” OR “Scaffold*” OR “Bone tissue engineering” OR “Tissue engineering”) AND (“Animal model*” OR “In vivo” OR “Animal study*” OR “Animal*”). 

The literature search was conducted until November 2021 from the following electronic databases: Ovid, Web of Science, PubMed and EBSCOHOST. In addition to that, any additional studies or published articles were searched manually by scanning the reference lists and hand-searching key journals. The search was limited to articles published in the English language and there was no defined time period on the year of publication.

### 2.2. Selection of the Studies

The initial screening of the title and abstracts was carried out independently by two researchers (N.M. and M.R.). Subsequently, the assessment of the eligibility of the full-text articles was made based on the inclusion and exclusion criteria. Any disagreement between the reviewers on study selection and data extraction was consulted with a third reviewer (N.H.A.K.) as needed. 

The inclusion criteria were defined according to Participant (P): animal models undergoing bone regenerative surgery in the oral cavity, Concept (C): the intervention to regenerate or augment bone using 3D-printed HA or other calcium phosphates, with or without a combination of other biomaterials, and Context (C): new bone formation and biological properties of 3D-printed HA-based or other calcium phosphates. The types of sources involved prospective experimental animal studies with and without the 3D-printed HA. The articles were excluded if they were in vitro studies, human studies, case reports, review papers and conference abstracts. Articles that were not related to oral bone defects were also excluded.

### 2.3. Data Extraction and Analysis

Extraction of the information from the included articles was summarized according to the research questions and objectives into a table of evidence. The data were initially extracted by the first researcher (N.M.) and verified by the second researcher (M.R.) to ensure accuracy. The data of interest included publication details (first author, year and country of the study), the study design characteristics (total sample size, sample descriptions, interventions), animal model characteristics (animal species, gender, age, weight, defect size), method of 3D printing, methods of biological assessment (histology, microcomputed tomography) and outcomes (new bone formation, cell viability, tissue reaction).

## 3. Results

### 3.1. Study Selection and Characteristics

**Figure 1:**
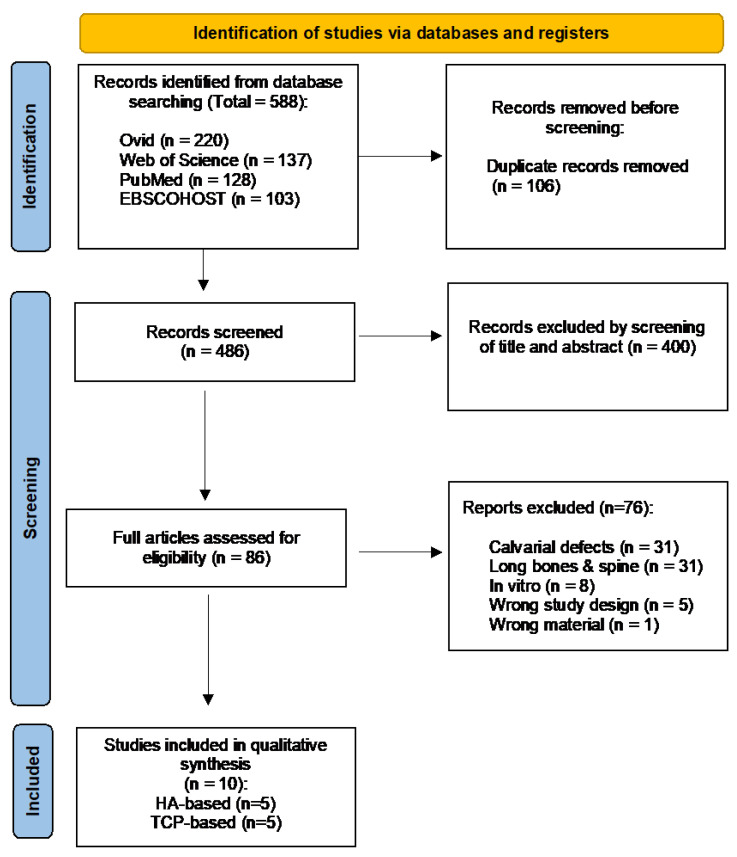
PRISMA flow diagram depicting the results of the search strategy.

A total of 588 records from 4 databases: Ovid (n = 220), Web of Science (n = 137), PubMed (n = 128) and EBSCOHOST (n = 103), were generated from the search strategy until November 2021. Out of these, 106 duplicates were removed and 486 were screened based on the titles and abstracts. Eighty-six articles were considered in the full-text screening eligibility based on the inclusion and exclusion criteria. Following the screening process, 76 articles were further excluded because the defects were not in the mandible or maxilla, calvarium (n = 31), long bones and spines (n = 31), in vitro study (n = 8), wrong study design (n = 5) and wrong material (n = 1). Finally, a total of 10 studies were included in this review, as recorded in the detailed flowchart in Figure 1.

### 3.2. Characteristics of Included Studies

The included studies followed the Animal Research: Reporting of In Vivo Experiments (ARRIVE 2.0) guidelines for quality assessment in this review [36]. The 10 selected articles were published between 2016 and 2021 and were conducted in the USA (n = 4) [37,38,39,40], Taiwan (n = 2) [41,42], Korea (n = 2) [43,44], China (n = 1) [45] and Switzerland (n = 1) [46]. Table 1 and Table 2 summarize the details of the included studies using 3D HA-based and 3D TCP-based scaffolds.

### 3.3. Study Design and Osseous Defects

Eight out of ten studies used large animal models, which were beagle dogs [43,44,46], canines [37] and rabbits [38,39,40,45], whereas the other two used small animal models, Sprague-Dawley rats, in their studies [41,42] (Table 3). Tooth extraction of lower premolars and molars was carried out with a healing period of 6 to 12 weeks prior to defects’ preparation [37,43,46]. Eight studies involved surgically created defects on the mandible [37,38,41,42,43,44,45,46] and the remaining two studies were on the maxilla [39,40] to receive either 3D-printed HA-based or TCP-based bone scaffolds.

Three studies reported a combination of four interventions: 3D-printed HA and RGD-functionalized alginate matrix (RAM) as a supplement [42], 3D-printed HA with additional features of microchannels or macro-channels with or without a screw [37] and 3D-printed TCP and calcium silicate porous bioceramics [45]. Six studies were designed to have negative control groups, which were unfilled or untreated bone defects [38,39,40,41,42,43], and one positive control group [43]. Two studies reported the blinding of the treatment assignment to the surgical procedures [41,42].

### 3.4. Three-Dimensional-Printed HA- and TCP-Based Bone Scaffolds

Three studies reported using biodegradable synthetic polymer that acts as a binder, 3D-printed HA and poly(lactic-co-glycolic acid) (PLGA) [41,42] and 3D-printed TCP and PCL [44]. The other two studies used combinations of tricalcium phosphates as the experimental materials, which were α-TCP and calcium-deficient HA (CDHA) (OsteoFlux^®^, Vivos-Dental, Villaz-St-Pierre, Switzerland) [46], and HA and TCP [43]. Four articles documented using β-TCP only [38,39,40,45] and one article used plain HA (TheriRidge) as bone scaffolds [37]. There was only one study reporting the application method of the adipose stem cells’ seeding prior to implantation of the 3D-printed TCP-based scaffolds [44]. 

Three studies used the extrusion technique to fabricate the 3D-printed HA bone scaffolds [41,42,46]. Both studies by Chang et al. used an extrusion-based 3D printer (INKREDIBLE Cell Inc, Gothenburg, Sweden) with the following settings: 100 to 250 kPa, speed of 1 mm/s and 0.3 mm layer thickness [41,42]. Another two studies reported producing 3D-printed HA scaffolds by using the digital light processing (DLP) stereolithography technique [37,43]. A 3D printer (Cubicon Lux, Cubicon^®^, Sungnam, Korea) with a high resolution of 100 μm was used in the study by Kim et al. [43]. For 3D-printed TCP bone scaffolds, a total of four studies reported using direct-writing 3D printers. Two of the studies used Aerotech Inc., Pittsburgh, PA, USA [38,39], one study used 3D Inks LLC [40], however study by Shao et al did not report specific brand of the 3D printer [45]. After printing, scaffolds were sintered at temperatures ranging from 400 to 1150 °C to densify the constructs and remove impurities [47]. Another study by Lee et al. used a fused deposition system for fabricating the 3D-printed TCP-based bone scaffolds [44].

Following implantation of the graft to the surgically created alveolar bone defects, the defect sites were either covered the with collagen membrane (Biogide^®^, Geistlich, Switzerland) only [46], collagen membrane (Genoss^®^, Suwon, Korea) and fixation pins (Dentium^®^, Suwon, Korea) [43], with fixation screws (KLS Martin) [37], or with plates and screws (Signo-Vinces, Campo Largo, Brazil) [38,44] (Table 4). The time of the animals’ euthanization after implantation varies from day 7 to 18 months depending on the required analysis in the studies.

### 3.5. Study Outcome Measures

#### 3.5.1. Clinical Evaluation

All animals in this review survived during the experimental period until sacrifice. However, one study had failure of the two-blocks graft because of the soft tissue dehiscence [37] and another article reported that the resorbable collagen membrane overlying the bone graft material was not degraded completely at four or eight weeks [43]. Another study reported that two animals had post-operative infection after the surgery and the samples were excluded from the analysis [38].

#### 3.5.2. Measurement of the Bone Regenerative Outcomes

Nine out of ten studies used histological assessment, which was the most common measurement of the bone regeneration outcomes in alveolar bone, that evaluated the inflammatory response of soft tissue, osteogenesis characteristics and bone ingrowth [37,38,39,40,41,42,44,45,46]. Apart from that, another evaluation of histomorphometric analysis was reported in four studies for quantitative measurement of the bony ingrowth, neovascularization and 3D-printed block resorption [37,43,45,46]. For the imaging method, microcomputed tomography [38,39,40,41,42,43,44,45] was used for the analysis of bone regeneration in the mandible and maxilla in vivo. Three studies used gene expression for osteogenic differentiation during the healing stage [41,42,44] and one study evaluated the expression of proteins related to ossification during implantation of the 3D-printed HA- and TCP-based scaffolds [44].

#### 3.5.3. Bone Regenerative Outcomes of the 3D-Printed HA- and TCP-Based Scaffolds

**Figure 2:**
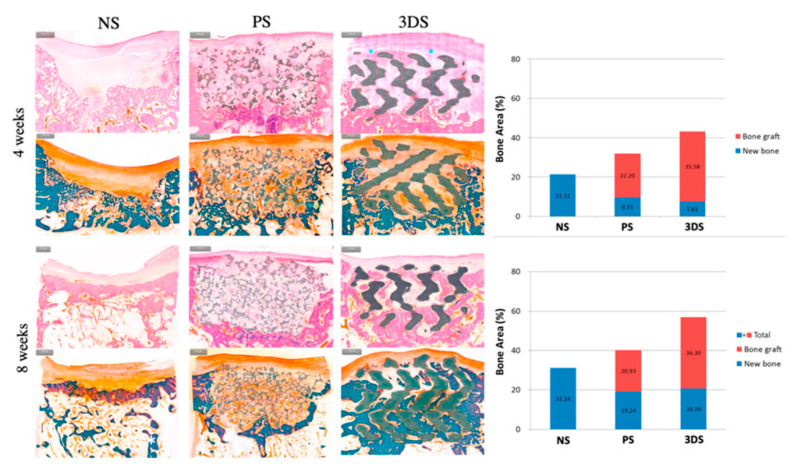
Histomorphometric evaluation at 4 and 8 weeks. The 3DS group showed a higher value of total amount of bone than PS and NS groups at 4 weeks. At 8 weeks, the greatest total amount of bone was in the 3DS group, followed by the PS and NS groups. 3DS: 3D-printed HA/TCP; PS: Particle-type substitute (OSTEON 3, Genoss^®^, Suwon, Korea); NS: Untreated defect [43].

The histological assessment of the mandible showed the formation of new bone in a defect filled with 3D-printed HA/PLGA [41,42] and higher ossification in 3D-printed TCP/PCL coated with bdECM and seeding of ADSCs [44]. The other studies also reported an increased amount of bone ingrowth in 3D-printed α-TCP and CDHA [46] and 3D-printed plain HA [37], and 3D-printed β-TCP only [38,39,40,45]. Another regenerative outcome from the histomorphometric analysis showed that the 3D-printed HA scaffolds can induce new bone and vasculature formation in osseous defects in vivo [37,43,46], except one study by Shao et al. reported that 3D-printed β-TCP scaffolds had the lowest new bone formation and the highest relative residual material compared to the bioceramic scaffolds during the implantation period [45]. Figure 2 showed histomorphometric evaluation of the 3D-printed HA-based scaffolds.

**Figure 3:**
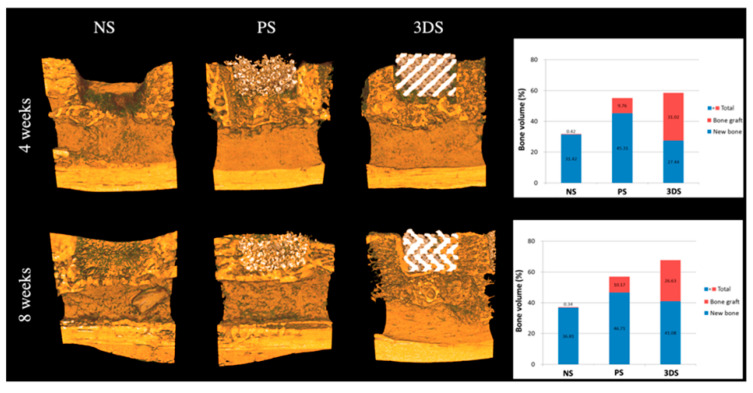
Radiological evaluation at 4 and 8 weeks. The 3DS and PS groups showed greater values of the total amount of bone than the NS group at 4 weeks. There were no significant differences in the amounts of new bone at 8 weeks among all the groups. The three groups from the highest to least relative total amounts of bone were: 3DS, PS and NS. 3DS: 3D-printed HA/TCP; PS: Particle-type substitute (OSTEON 3, Genoss^®^, Suwon, Korea); NS: Untreated defect [43].

The micro-CT assessment showed that the mineralized tissue was greater in defects filled with 3D-printed HA scaffolds compared to the unfilled defect, which was deposited with newly formed mineralized tissue as early as the fourth week [41]. The finding from the group of 3D-printed HA with the oxidized RAM supplement showed a greater bone volume/radiographic region of interest [42]. The study by Kim et al. also reported an increased total amount of bone observed in the 3D-printed HA/TCP group compared to the untreated group [43] as shown in Figure 3. 3D-printed β-TCP-based scaffolds, coated with either DIPY, rhBMP-2 or bdECM, also reported new bone tissue ingrowth at the osseous defect [38,39,40,44,45]. For early gene expression in the mandibular osseous defects in vivo, 3D-printed HA/PLGA showed significant upregulation of vascular endothelial growth factor (VEGF) and RUNX2 in the 3D group with oxidized RAM supplement [42], and type 1 collagen (Colla1), VEGF and core-binding factor alpha-1 (Cbfa1) relative to the control group [41]. A study by Lee et al. reported greater expression of genes and proteins related to ossification, which were Colla1, osteocalcin and RUNX2 in 3D-printed β-TCP/PCL coated with bdECM and ADSCs aggregates [44].

## 4. Discussion

Three-dimensional printing technology is a promising alternative for the fabrication of scaffolds for bone regeneration in the oral cavity. The included studies showed various aspects of heterogeneity in the combination of HA and TCP materials, study design, bone defect characteristics, measurement of outcomes and animal models. This review focused on bone tissue engineering using 3D-printed HA- and TCP-based scaffolds with and without polymers that have been developed as alternatives to autografts to repair bone defects in vivo. HA has similar mineral components as bone tissue and superior osteoconductivity compared to other calcium phosphates, making HA the material of choice in bone regeneration research [48]. Biocomposite materials made from combining HA with biodegradable polymers, both natural and synthetic, may provide good results on bone regeneration, with both materials benefitting from each other’s constituents [49]. With the combination of 3D-printed HA and synthetic polymer, PLGA demonstrated favorable osteoregenerative potential in critical-sized defects [41,42]. Asa’ad et al. reported that PLGA has been a popular polymer in tissue engineering due to its mechanical stability, low degradation rate, moldable and biocompatible material [50]. However, its major disadvantages, such as a lack of intrinsic bioactivity, might be solved by incorporating HA to establish bioactive interfaces. The combination of the 3D-printed HA scaffold (90% in weight) and PLGA (around 10%) reported good elastic characteristics and absorbent capacity, which is acceptable for bone cells’ activities [51].

Another three studies did not use a polymer in 3D-printed HA scaffolds [37,43,46], and it has shown the potential of bone regeneration with the combination of 3D-printed HA and TCP. However, studies reported that the combination of HA/TCP with polymer has inferior results in terms of new bone-forming potential and biocompatibility because of the polymer addition [52,53]. The combination of HA/TCP is known to exhibit excellent osteoconductivity and osteoinductivity properties, biocompatible and biodegradable material [54,55], and β-TCP is the most desirable form of TCP scaffold due to its chemical stability and mechanical strength [43,56]. The composition of BCP (HA/β-TCP ratio) is an important parameter for the healing outcome of bone defects [57]. The ratio must deliver the balance between the mechanical stability which has been provided by HA and the dissolution rate of β-TCP depending on the location of bony defects [57]. A study by Petrovic et al. reported that the degradation rate of BCP is higher than HA but slower than β-TCP [58]. This controllable degradation rate is an important feature of BCP [59] in order to achieve complete bone formation. The higher ratio of HA to β-TCP is suitable for bone regeneration in dehiscence types of defects and surgically created periodontal defects, as used by Kim et al. with a combination of HA/TCP (6:4 ratio) [43]. The greater the HA composition, the longer stability of the implanted graft to constant stress during mastication [60,61,62]. Another type of calcium phosphate group is calcium-deficient hydroxyapatite (CDHA) scaffolds, which can be obtained by hydrolysis of α-TCP and have a calcium to phosphate molar ratio lower than 1.67 [63]. The 3D-printed CDHA showed vertical bone growth from the bone bed, as in Carrel et al.’s work [46]. Several studies also reported that the CDHA scaffold was able to induce reparative cells to repair a bone defect, faster biodegradation and better osteoinductivity properties compared to HA [64,65,66].

Porosity, pore size and interconnected network are imperative design characteristics of the 3D-printed scaffold. 3D printing technology can have high precision in terms of controlling the porous structure and the porosity of the scaffolds [67]. Three included studies reported that the total porosity of 3D-printed HA- and TCP-based bone scaffolds were from 38% to 65% [42,45,46], which is within the range of 30% to 90% of human cancellous bone [68]. Regarding the pore size, the dimension > 300 μm has been reported to be optimal for bone tissue engineering in vivo [26] and five selected studies used a pore size of 330 to 500 μm for regeneration of osseous defects in the mandible [38,41,42] and maxilla [39,40]. A higher amount of porosity and pore size will reduce the mechanical stability of the scaffolds. The interconnectivity of the pore network is also important in tissue-engineered constructs [67] for cell growth into the interior part of the scaffolds.

The heterogeneity of the included studies in treatment modalities makes it difficult for clinicians or researchers to delineate the outcomes. All studies involved surgical preparation of the sites in the mandible either for the intervention used or restoring critical-sized defects. The critical-sized defect is the defect which is unable to heal spontaneously or regenerate itself and it requires the use of graft material to guide the action of bone-regenerating cells [15,69]. The intervention studies which involved guided bone regeneration treatment and bone augmentation were mostly covered by the collagen membrane [43,46], fixing pin [43], plate and screw [38,44] or screw [37]. Two studies used the through and through jaw bone defect, which is a critical-sized defect of the mandibular ramus, for evaluation of the 3D-printed HA bone scaffolds [41,42]. All the results of the 3D-printed HA- and TCP-based bone scaffolds exhibited new bone growth and were biocompatible to the tissues which can be a potential alternative material for the autogenous bone graft.

The measurement of the studies’ outcomes was commonly assessed using histological, histomorphometric and computed tomography techniques. Micro-CT imaging is able to provide a description of 3D outcomes for trabecular bone microarchitecture, such as bone volume fraction, trabecular number, thickness and separation [70]. Mineralized tissue of bone scaffolds and newly formed bone were reported in [38,39,40,41,42,43,44,45]. However, this assessment limits the ability to differentiate the marrow space and the soft tissue. Therefore, the histological and histomorphometry analyses could provide information on cell types and soft tissue response. 

Histomorphometry remains the most widely used technique to evaluate the bone microarchitecture, bone formation and bone remodeling through different cell type activities [71]. Even though this technique affords a high resolution and a good image contrast, it remains time-consuming and expensive [72]. This quantitative assessment evaluates two-dimensional (2D) data, which focus on limited sections from the whole bone volume and do not reflect the actual 3D structure of bone [72]. In spite of the limitations, histological analysis is still required to validate the imaging results and provide information on the quality of new bone and formal assessment of mineralization [73]. In this review, two studies did not use any imaging assessment for the intervention outcomes and only depend upon histology and histomorphometric analyses [37,46]. The combination of information from imaging (3D analysis) and histology (2D analysis) is essential to evaluate the osteogenic potential in the animal model [41]. Another assessment that has been mentioned in the included studies was early gene expression for osteogenic differentiation in the osseous defect [41,42]. The early genes, such as type 1 collagen (Colla1), vascular endothelial growth factor (VEGF), core-binding factor alpha-1 (Cbfa1) and RUNX2, are important genes for the evaluation of the initiation of matrix synthesis, angiogenesis and osteogenic differentiation at an early healing stage [74,75,76].

Generally, several different animal models are used in investigating bone regeneration in the oral cavity, which include dogs, pigs, mini-pigs, rabbits, sheep and rodents. For evaluation of the biological properties of the biomaterials, a suitable animal model needs to be established. The main factor that the researcher needs to consider when choosing the animal species is the differences in anatomy and physiology of the animal to human bone [77,78]. Three studies used dogs as large animal models reported in this review. In bone tissue regeneration, the dog model has been used extensively because of the comparable bone metabolism and alveolar bone defect healing process to humans [79]. Dogs will reach skeletal maturity at the age of 10 to 18 months, with an adult weight of 15 to 30 kg [77,78]. One study conducted an experiment on immature beagle dogs as their animal model [43]. The optimal study design should consider the use of skeletally mature animals of an appropriate age because of the difference in healing potential and response [80]. Larger animals such as dogs or monkeys have well-developed Haversian remodeling compared to rats [81]. Two of the studies in this review used Sprague-Dawley rats weighing 250–300 g [41,42], which is equivalent to the adult weight [78]. The advantage of using pre-clinical rat models for critical-sized defects is to produce a proof-of-concept study in order to establish ideal novel bone scaffolds in a short timeframe [82]. Rats and mice are among the popular selection of small animal models because of their size, cheaper cost and their ease of handling [83]. However, the limitation of using these animals is their macroscopic dissimilarity to the human bone [77], and the findings from small animal models often do not translate into human clinical applications [84,85]. The difference in regenerative techniques and the sacrifice times in between studies varied from 1 week [41,42] to 26 weeks [37], which contributed to a great variation of the observation window in the animal models. In addition to that, there were limited 3D-printed HA- and TCP-based bone scaffold in vivo studies on the mandible or maxilla compared to calvarium and long bones, which make the comparison of bone regeneration between studies difficult despite being in the same animal model.

There are several limitations in this scoping review. Firstly, there were two different research models used, which were associated with and without physiological loading, and these could have an impact in terms of simulation to the dentoalveolar environment. Secondly, the nature and pattern of the surgically created defect might not completely reflect the true condition of the disease and the alveolar ridge. Hence, the fabrication of customized scaffolds could be established in a clinically relevant osseous defect in future studies. Finally, the treatment approaches and outcome parameters assessed in the included studies vary widely, which makes it impossible for a direct comparison of materials and treatment groups that have superior bone regeneration outcomes in animal models.

## 5. Conclusions

This review reported that the 3D-printed HA- and TCP-based bone scaffolds are safe and biocompatible, and demonstrated bone regenerative capabilities in the oral cavity. Despite the regeneration potential of 3D-printed HA- and TCP-based bone scaffolds for intraoral bony defects, there is still inadequate evidence to substantiate which material is congruent with the gold standard of care. Therefore, future research should simulate the autogenous bone block by exploiting the cell scaffold-based approach, combined with different types of biodegradable polymers that can be utilized for bone regeneration. Research should also focus on the regenerative potential of 3D-printed bone scaffolds in the load-bearing areas, including the masticatory load of the oral cavity.

## Figures and Tables

**Table 1:** Summary of the included studies using 3D HA-based scaffolds.

Author	3D Material (Test)	Used Supplement	3D Printing Technique	Assessment Methods	Main Findings
Carrel et al., 2016 [46]	α-TCP and micro-crystalline/ CDHA (OsteoFlux)	-	Extrusion	Histology, Histopathology, Histomorphometry	New bone growth above its natural bed up to 4.5 mm
Fiorellini et al., 2018 [37]	HA (TheriRidge)	-	Digital light processing (DLP)	Histopathology, Histomorphometry	3D-printed blocks exhibit new bone growth adjacent to and within the graft. The amount of bone ingrowth and the presence of osteoid were slightly higher in the blocks without a screw
Kim et al., 2020 [43]	HA/TCP (6:4 ratio) (Genoss)	-	Digital light processing (DLP) stereolithography	Micro-CT, Histomorphometry	Total amount of new bone formation higher in 3D HA scaffold than particle-type substitute
Chang et al., 2021 [41]	90 wt.% HA/10 wt.% 82:18 PLGA	-	Micro- extrusion	Gene expression, Micro-CT, Histology	Allow direct bone apposition and facilitate new bone formation compared to the control group
Chang et al., 2021 [42]	90 wt.% HA/10 wt.% 82:18 PLGA	RGD-functionalized alginate matrix (RAM)	Micro- extrusion	Gene expression, Micro-CT, Histology	Adding oxidized RAM with osteoid-like stiffness induces bone formation and facilitates the synthesis of collagen, angiogenesis and osteogenesis

TCP, Tricalcium phosphate; HA, Hydroxyapatite; CDHA, Calcium-deficient hydroxyapatite; PLGA, poly(lactic-co-glycolic acid); RAM, RGD-functionalized alginate matrix; Micro-CT, Microcomputed tomography.

**Table 2:** Summary of the included studies using 3D TCP-based scaffolds.

Author	3D Material (Test)	Cells Seeding	3D-Printing Technique	Assessment Methods	Main Findings
Lopez et al., 2018 [38]	β-TCP	-	Direct writing	Micro-CT, Histology	β-TCP scaffolds able to repair critical segmental mandibular defects to levels similar to native bone
Shao et al., 2018 [45]	β-TCP	-	Direct writing	Micro-CT, Histology, Histomorphometry	β-TCP had lowest new bone formation compared to other materials (CSi, CSi-Mg10 and Bred)
Lopez et al., 2019 [39]	β-TCP (coated with DIPY or rhBMP-2)	-	Direct writing with additive manufacturing	Micro-CT, Histology	Both β-TCP scaffolds with DIPY or rhBMP-2 able to regenerate vascularized bone in skeletally immature alveolar bone defects
Lee et al., 2021 [44]	PCL/β-TCP (coated with bdECM)	ADSCs	Fused deposition	Micro-CT, Histology, Gene expression, Protein expression	PCL/TCP coated with bdECM and ADSC aggregates increased mandibular ossification
Shen et al., 2021 [40]	β-TCP (coated with DIPY)	-	Direct writing	Micro-CT, Histology	β-TCP/DIPY scaffolds accelerate degradation rate and replacement of β-TCP with vascularized bone

TCP, Tricalcium phosphate; PCL, Polycaprolactone; bdECM, bone demineralized and decellularized extracellular matrix; CSi, Wollastonite; CSi-Mg10, ~10% magnesium-substituted wollastonite; Bred, Bredigite; DIPY, Dipyridamole; rhBMP-2, recombinant human bone morphogenetic protein-2; ADSC, Adipose-derived stem cells; Micro-CT, Microcomputed tomography.

**Table 3:** Summary of animal model characteristics.

Author	Animal Model	Total No of Defects	Sex	Age	Weight	Defect Size	Time of Sacrifice
Carrel et al., 2016 [46]	Beagle dogs	4	Male	18 months	16 kg	-	8 weeks
Fiorellini et al., 2018 [37]	Canines	32	Male	NR	NR	8 × 5 mm	16, 26 weeks
Kim et al., 2020 [43]	Beagle dogs	48	Male	22–26 weeks	10–12 kg	7 × 3 × 5 mm^3^	4, 8 weeks
Chang et al., 2021 [41]	Sprague Dawley rats	28	Male	NR	250–300 g	4 mm (diameter)	1, 4 weeks
Chang et al., 2021 [42]	Sprague Dawley rats	60	Male	NR	250–300 g	4 mm (diameter)	1, 4 weeks
Lopez et al., 2018 [38]	NZ white rabbits	8	NR	NR	~3.5 kg	12 mm	8 weeks
Shao et al., 2018 [45]	NZ white rabbits	64	Male	NR	2.8 ± 0.2 kg	10 × 6 × 4 mm^3^	8, 16 weeks
Lopez et al., 2019 [39]	NZ white rabbits	24	NR	NR	NR	3.5 × 3.5 mm	8 weeks
Lee et al., 2021 [44]	Beagle dogs	10	NR	36 months	NR	-	8 weeks
Shen et al., 2021 [40]	NZ white rabbits	22	NR	1 month	NR	3.5 × 3.5 mm	2, 6, 8 and 18 months

NZ, New Zealand; NR, Not Reported.

**Table 4:** The characteristics of 3D-printed HA- and TCP-based bone scaffolds and study interventions.

Author	3D-Printed Scaffolds (Test)	Additional Features to 3D-Printed Scaffolds	Porosity/Pore Size	Pre-Intervention	Intervention	Additional Material to Cover/Fix 3D-Printed Scaffolds
Carrel et al., 2016 [46]	α-TCP and microcrystalline/CDHA (OsteoFlux)	Regular porosity and forms an interconnected network, scaffold’s macro-porosity 40% to 50%	Total porosity 50% to 65%	Extraction of mandibular first premolar to the first molar (both sides)	Guided bone regeneration	Collagen membrane
Fiorellini et al., 2018 [37]	HA (TheriRidge)	Macro-channel blocks with through and through mesial to distal channel (1.4 × 1.6 mm) or microchannel blocks with through and through buccal to lingual channel (20–50 μm)	NR	Extraction of mandibular first premolar to the first molar (both sides)	Alveolar ridge augmentation	Fixation screw
Kim et al., 2020 [43]	HA/TCP (6:4 ratio) (Genoss)	-	NR	Extraction of mandibular first premolar to the first molar (both sides)	Guided bone regeneration	Collagen membrane and fixation pins
Chang et al., 2021 [41]	90 wt.% HA/10 wt.% 82:18 PLGA	Orthogonal pores	Pore size 400 × 400 μm Mean pore size of 0.420 ± 0.028 × 0.328 ± 0.005 mm^2^	-	Regeneration of mandibular critical-sized defects	-
Chang et al., 2021 [42]	90 wt.% HA/10 wt.% 82:18 PLGA	Interconnected orthogonal pores with lid (6 mm diameter) to hold main body for the scaffold	Total porosity 37.78% ± 2.30% Pore size 400 × 400 μm Mean pore size 0.426 ± 0.041× 0.368 ± 0.015 mm^2^	-	Regeneration of mandibular critical-sized defects	-
Lopez et al., 2018 [38]	β-TCP	-	Pore spacing 330 μm	-	Regeneration of mandibular critical-sized defects	Plate and screws
Shao et al., 2018 [45]	β-TCP	-	Total porosity 57.3% ± 4.4% Pore size 302 ± 17.2 × 261 ± 12.9 μm	-	Regeneration of alveolar bone defect	-
Lopez et al., 2019 [39]	β-TCP (coated with DIPY or rhBMP-2)	-	Pore spacing 330 μm	-	Regeneration of alveolar bone defect	-
Lee et al., 2021 [44]	PCL/β-TCP (coated with bdECM)	4 holes, diameter 1 mm	NR	Extraction of mandibular first premolar to the first molar (left side)	Mandibular reconstruction	Plate and screws
Shen et al., 2021 [40]	β-TCP (coated with DIPY)	-	Pore spacing 500 μm	-	Regeneration of alveolar bone defect	-

TCP, Tricalcium phosphate; HA, Hydroxyapatite; CDHA, Calcium-deficient hydroxyapatite; PLGA, poly(lactic-co-glycolic acid); RAM, RGD-functionalized alginate matrix; PCL, Polycaprolactone; bdECM, bone demineralized and decellularized extracellular matrix; DIPY, Dipyridamole; rhBMP-2, recombinant human bone morphogenetic protein-2; NR, Not Reported.
